# Fulminant meningococcal supraglottitis: An emerging infectious syndrome?

**DOI:** 10.3201/eid0503.990323

**Published:** 1999

**Authors:** E Schwam, J Cox

**Affiliations:** Sturdy Memorial Hospital, Attleboro, Massachusetts 02703-7043, USA. schwann@massmed.org

## Abstract

We report a case of fulminant supraglottitis with dramatic external cervical swelling due to associated cellulitis. Blood cultures were positive for Neisseria meningitidis. The patient recovered completely after emergency fiberoptic intubation and appropriate antibiotic therapy. We summarize five other cases of meningococcal supraglottitis, all reported since 1995, and discuss possible pathophysiologic mechanisms.


### Fulminant Meningococcal Supraglottitis: An Emerging Infectious Syndrome?

**Eric Schwam*† and Jeffrey Cox†**
*Sturdy Memorial Hospital, Attleboro, Massachusetts, USA; and
†Rhode Island Hospital, Providence, Rhode Island, USA

We report a case of fulminant supraglottitis with dramatic external cervical swelling due to associated cellulitis. Blood cultures were positive for *Neisseria meningitidis.* The patient recovered completely after emergency fiberoptic intubation and appropriate antibiotic therapy. We summarize five other cases of meningococcal supraglottitis, all reported since 1995, and discuss possible pathophysiologic mechanisms.

*Neisseria meningitidis*, a gram-negative diplococcus, can cause a broad spectrum of clinical manifestations including acute meningitis, meningococcemia, occult bacteremia, meningoencephalitis, pneumonia, conjunctivitis, dermatitis-arthritis syndrome, and urethritis (1). We describe a rare case of meningococcal supraglottitis (2-6) further complicated by cervical cellulitis. Reports of five other cases suggest an emerging clinical syndrome due to this pathogen.

### Case Report

In January 1998, a 44-year-old woman became ill with rhinitis and sore throat, which progressed to dysphagia, dyspnea, and neck swelling during the night, requiring her to sleep sitting upright. The following morning, she went to a community hospital emergency room.

The patient was alert but appeared toxic and had inspiratory stridor and a muffled voice. She had routine dental cleaning 1 week before onset of symptoms. She did not have a toothache and said she did not use tobacco or alcohol. She had had no history of splenectomy or immune deficiency. Her temperature was 38.1°C, pulse 138, cuff blood pressure 120/74 mm Hg, and respiratory rate 24. Room air pulse oximetry was 94%. The tongue was large, but not edematous, and there was neither drooling nor sublingual swelling. The soft palate and posterior pharynx, seen only with difficulty, were diffusely swollen, protruding anteriorly, and covered with exudate. Massive external swelling, tenderness, and erythema of the anterior neck extended from the chin caudad to the midsternum, obliterating all cervical landmarks. The patient did not have meningismus, crepitus, or jugular venous distention. The lungs and heart were normal. No rash was present.

A portable lateral radiograph of the neck showed diffuse soft tissue cervical and epiglottic swelling with a classic "thumb sign." White blood count was 21.9 $\times 10^9$/L with 0.70 polymorphonuclear leukocytes and 0.17 bands. Platelet count was 242 $\times 10^9$/L. Hematocrit, electrolytes, glucose, and urea nitrogen were normal. The patient was treated with oxygen and nebulized epinephrine, ceftriaxone 1.0 gram intravenously (i.v.), and clindamycin 600 mg i.v. She was taken to the operating room, where with surgical standby, she was orally intubated with a 6.0 mm-endotracheal tube over a fiberoptic laryngoscope.

The patient was transferred to a tertiary care hospital, where the antibiotics were changed to ampicillin-sulbactam, clindamycin, and gentamicin. Computed tomography (CT) scan of the neck and chest showed extensive soft tissue swelling from the oropharynx to the supraglottic region with obliteration of the airway surrounding the endotracheal tube. The adjacent parapharyngeal and cervical soft tissues down to the upper chest were also involved, but no discrete abscess was identified. Bilateral pulmonary infiltrates, atelectasis, and small pleural effusions were present, but the mediastinum was normal.

Address for correspondence: Eric Schwam, Emergency Care Center, Sturdy Memorial Hospital, 211 Park Street, Attleboro, MA 02703-0963, USA; fax: 508-236-7043; e-mail: schwam@massmed.org.

The next day, two blood cultures drawn before administration of antibiotics grew *N. meningitidis,* serogroup Y (confirmed by the Massachusetts Department of Public Health Laboratory). The organism was sensitive to penicillin and ceftriaxone and resistant to tetracycline by the Kirby-Bauer method. Sputum culture done by endotracheal tube (after the start of antibiotics) was negative. Close family contacts and the community hospital staff members involved with airway procedures were given prophylactic antibiotics, according to guidelines (rifampin 600 mg PO bid x 4 doses, ciprofloxacin 500 mg PO x 1 dose, or ceftriaxone 250 mg intramuscularly x 1 dose) (7).

Clindamycin and gentamicin were discontinued. Repeat CT scan on hospital day 5 was unchanged, but by day 7, the swelling had resolved, and the patient was extubated in the operating room. She recovered fully and was discharged on hospital day 9; she received i.v. ampicillin-sulbactam at home for an additional 7 days. She remained well, as determined by telephone contact 13 months later. Complement testing (CH-50) 13 months later was normal.

### Conclusions

The diagnosis of supraglottitis is most consistent with the patient's clinical picture of sore throat, dysphagia, fever, muffled voice, and swollen supraglottic tissues, as seen on plain films, fiberoptic laryngoscopy, and cervical CT scan (8). However, supraglottitis is uncommonly accompanied by such dramatic external cervical swelling. Ludwig's angina was initially considered, but this diagnosis was unlikely because odontogenic infection and involvement of the sublingual or submandibular spaces were lacking. Cervical abscesses can complicate supraglottitis (9), but no abscess was detected on repeated CT scans. Necrotizing fasciitis was a possibility, especially since the erythema and swelling overlying the chest suggested mediastinal extension. In the one reported case of supraglottitis with cervical necrotizing fasciitis (10), fascial gas was demonstrated on CT scan. Surgical drainage was required for cure; anaerobic organisms were the likely cause. Furthermore, *N. meningitidis,* which rarely causes cellulitis, has not been reported to cause necrotizing fasciitis. The pathologic process in bacterial supraglottitis is cellulitis of the epiglottis and the surrounding upper airway. In this patient, the cellulitis was so aggressive that it extended posteriorly to the spine, anterolaterally to the cervical skin, cephalad to the pharynx, and caudad to the chest.

Cultures of the epiglottis and pharynx were not performed, so the meningococcal bacteremia may have reflected secondary or coincident infection, not supraglottic infection. However, even when laryngeal cultures are performed, organisms isolated from the blood and the throat correlate poorly (11). Although the CT lung scan demonstrated pulmonary infiltrates, bacteremic meningococcal pneumonia complicating supraglottitis due to another organism is improbable.

A search of Medline (the National Library of Medicine's bibliographic database of biomedical journals) from 1966 through mid-1999 located five reports of meningococcal supraglottitis: two from Colorado (2,4), one from Ohio (3), one from Singapore (5), and one from Helsinki, Finland (6). Including our patient in the series of six, the ages of the patients (three were women) were 44, 54, 60, 65, 81, and 95. Two patients had type 2 diabetes mellitus, but the others were otherwise healthy; all had fever, sore throat, and evidence of upper airway compromise. No upper airway cultures were reported, but blood cultures in all six cases were positive for *N. meningitidis*: serogroup B (two), serogroup Y (three), and unreported serogroup (one). No clinical evidence of meningitis or meningococcemia was found in any of the cases. Only our patient had external cervical cellulitis. All patients recovered with appropriate antibiotic treatment. The possibility of complement deficiency was investigated and ruled out in two of two patients. Two patients were treated with steroids i.v. Five of the six patients required airway intervention (three, intubation; two, urgent tracheostomy), a much higher proportion than that in a population-based case series of adults with supraglottitis (11). Thus, bacteremic meningococcal supraglottitis appears to be fulminant and life-threatening.

Most meningococcal disease in the United States is sporadic. One third of all cases occur in adults, who are usually immunocompromised (e.g., by complement deficiency, corticosteroid use, or HIV infection). In one population-based study, more than half the adults had neither rash nor meningitis. Pneumonia, sinusitis, and tracheobronchitis were the main sources of bacteremic meningococcal disease. Supraglottitis was not observed (12). In particular, the proportion of meningococcal infections due to serogroup Y has been increasing nationally in the last several years. Serogroup Y is frequently associated with meningococcal pneumonia in both civilian (13) and military populations (14).

Why *N. meningitidis* is not a more frequent cause of supraglottitis is not known. The organism is a common colonizer of the upper airways of healthy persons, and the pharynx is the suspected portal of entry of invasive and disseminated disease (1). The organism may also cause simple pharyngitis (15).

The pathogenic determinants of both meningococcal disease and supraglottitis are complex and largely undefined, so we can only speculate how meningococci might cause this syndrome. Meningococcemic syndromes seem to require both epithelial and endothelial invasiveness so that the organism can cross the nasopharyngeal mucosal barrier, enter the bloodstream, and invade other blood vessel walls to produce the characteristic vasculitic organ damage. In contrast, meningococcal isolates from supraglottic syndromes seem to have relatively greater epithelial invasiveness, a propensity for contiguous local inflammatory spread, and decreased tropism for endothelial cells; these characteristics result in a more locally aggressive but less disseminated disease. The presence of various surface-expressed virulence factors (e.g., capsule, pili, cell surface proteins, and lipo-oligosaccharides) that mediate the organism's interaction with certain host cells may explain these differing pathophysiologic properties. For example, certain Opa cell surface proteins facilitate invasion of epithelial cells, while Opc proteins are more efficient at promoting invasion of endothelial cells (16).

*N. meningitidis* can cause an inflammatory conjunctivitis that progresses to septicemia in approximately 10% of cases (17). In an analogous manner, it can (rarely) extend locally to produce a periorbital cellulitis. In one such case, isolates from the blood and periorbital aspirate of the same patient were identical except for their expression of Opa proteins and their lipo-oligosaccharide phenotype (18).

Host factors (e.g., specific immune system deficiencies) could also be responsible for different disease manifestations. The most well-known example is the susceptibility of patients with terminal complement component deficiencies to neisserial infections. However, these patients have recurrent, but typical meningococcemia, so this deficiency would not be expected to contribute to the supraglottitis syndrome (19).

While *N. meningitidis* has been known for nearly 2 centuries and blood cultures have been routinely available for decades, meningococcal supraglottitis had not been reported until 1995 (2). In contrast, incidence of supraglottitis due to *Haemophilus influenzae* has remained constant in adults 18 years of age or older (11). Furthermore, while one case was reported each year from 1995 to 1997, three cases have been reported in 1998-1999, from three continents, suggesting the emergence of a new meningococcal syndrome worldwide. Surveillance is needed to determine if meningococcal supraglottitis will become more than just a rarity.

### Acknowledgment

We thank Dr. Gregory Jay for review of the manuscript.

Dr. Schwam practices emergency medicine at Sturdy Memorial Hospital in Attleboro, Massachusetts. He is clinical instructor in medicine at Brown University Medical School and instructor in emergency medicine at the University of Massachusetts Medical School.

Dr. Cox practices emergency medicine at Rhode Island Hospital in Providence, Rhode Island. He is assistant clinical professor of surgery at Brown University Medical School.

### References

1. Apicella MA. *Neisseria meningitis*. In: Mandell GL, Bennett JE, Dolin R, editors. Mandell, Douglas and Bennett's principles and practice of infectious diseases. 4th ed. New York: Churchill Livingston Inc.; 1995. p. 1896.
2. Crausman RS, Jennings CA, Pluss WT. Acute epiglottitis in the adult caused by *Neisseria meningitidis*. Scand J Infect Dis 1995;27:77-8.
3. Nelson K, Watkins DA, Watanakunakorn C. Acute epiglottitis due to serogroup Y *Neisseria meningitidis* in an adult. Clin Infect Dis 1996;23:1192-3.
4. Donnelly TJ, Crausman RS: Acute supraglottitis: when a sore throat becomes severe. Geriatrics 1997;52:65-6, 69.
5. Sivalingam P, Tully AM. Acute meningococcal epiglottitis and septicaemia in a 65-year-old man. Scand J Infect Dis 1998;30:198-200.
6. Mattila PS, Carlson P. Pharyngolaryngitis caused by *Neisseria meningitidis*. Scand J Infect Dis 1998;30:200-1.
7. Jafari HS, Perkins BA, Wenger JD. Control and prevention of meningococcal disease. MMWR Morb Mortal Wkly Rep 1997;46:RR-5.
8. Frantz TD, Rasgon BM, Quesenberry CP Jr. Acute epiglottitis in adults: analysis of 129 cases. JAMA 1994;272:1358-60.

9. Hebert PC, Ducic Y, Boisvert D, Lamothe A. Adult epiglottitis in a Canadian setting. Laryngoscope 1998;108:64-9.
10. Nguyen R, Leclerc J. Cervical necrotizing fasciitis as a complication of acute epiglottitis. J Otolaryngol 1997;26:129-31.
11. Mayo-Smith MF, Spinale JW, Donskey CJ, Yukawa M, Li RH, Schiffman FJ. Acute epiglottitis: an 18-year experience in Rhode Island. Chest 1995;108:1640-7.
12. Stephens DS, Hajjeh RA, Baughman WS, Harvey RC, Wenger JD, Farley MM. Sporadic meningococcal disease in adults: results of a 5-year population-based study. Ann Intern Med 1995;123:937-40.
13. Racoosin J, Diaz PS, Samala U. Serogroup Y meningococcal disease—Illinois, Connecticut, and selected areas, United States, 1989-1996. MMWR Morb Mortal Wkly Rep 1996;45:1010-4.
14. Koppes GM, Ellenbogen C, Gebhart RJ. Group Y meningococcal disease in United States Air Force recruits. Am J Med 1977;62:661-6.
15. Pether JVS, Scott RJD, Hancock P. Do meningococci cause sore throats? Lancet 1994;344:1636.
16. Nassif X, Magdalene SO. Interaction of pathogenic *Neisseriae* with nonphagocytic cells. Clin Microbiol Rev 1995;8:376-88.
17. Moraga FA, Domingo P, Barquet N, Glasser I, Gallart A. Invasive meningococcal conjunctivitis. JAMA 1990;264:333-4.
18. Patrick CC, Glen TF, Edwards M, Estabrook M, Blake MS, Baker CJ. Variation in phenotypic expression of the Opa outer membrane protein and lipooligosaccharide of *Neisseria meningitidis* serogroup C causing periorbital cellulitis and bacteremia. Clin Infect Dis 1993;16:523-27.
19. Figueroa J, Andreoni J, Densen P. Complement deficiency states and meningococcal disease. Immunol Res 1993;12:295-311.

